# Rheological Properties and Microscopic Mechanism of Nano-SiO_2_/SBS Composite Modified Asphalt

**DOI:** 10.3390/polym18161990

**Published:** 2026-08-15

**Authors:** Peng Yin, Baofeng Pan, Tianling Dong, Tao Liu, Shengkai Sun

**Affiliations:** 1 School of Infrastructure Engineering, Dalian University of Technology, No. 2, Linggong Road, Ganjingzi District, Dalian 116024, China; 2 Sichuan Provincial Communications Construction Group Co., Ltd., Chengdu 610041, China

**Keywords:** pavement engineering, modified asphalt, SBS composite modification, nano-SiO_2_, rheological properties

## Abstract

Asphalt serves as the core binder for heavy-load high-modulus pavements, and its viscoelasticity across a wide temperature range directly governs pavement-rutting resistance, low-temperature crack resistance and service life. Virgin asphalt contains abundant light fractions and exhibits insufficient stiffness at high temperatures. Modification with single styrene–butadiene–styrene block copolymer (SBS) fails to meet the anti-deformation requirements under heavy loads, while separate incorporation of nano-silica (nano-SiO_2_) aggravates low-temperature brittleness. Existing studies lack comprehensive investigations into the rheological evolution laws and synergistic microscopic mechanisms of asphalt modified by combined SBS and nano-SiO_2_. In this paper, virgin asphalt was adopted as raw material to prepare composite modified asphalt with gradient dosages. Integrated macroscopic performance tests and multi-scale microscopic characterizations were conducted for systematic analysis. High-temperature, low-temperature and fatigue performances were evaluated via conventional physical property tests, temperature sweep tests, multiple stress creep recovery (MSCR), linear amplitude sweep (LAS) and bending beam rheometer (BBR) tests. Fourier transform infrared spectroscopy (FTIR), gel permeation chromatography (GPC) and thin-layer chromatography–flame ionization detection (TLC-FID) were utilized to analyze variations in functional groups, molecular weight and four fractions, to elaborate the two-phase synergistic modification mechanism. The results demonstrate that the combined incorporation of SBS and nano-SiO_2_ synchronously optimizes the comprehensive performances of asphalt. Compared with single-SBS-modified asphalt, the sample with optimal dosages achieves elevated high-temperature modulus and rutting factor, reduced permanent deformation, improved low-temperature stress relaxation capacity and remarkably decelerated fatigue damage accumulation rate. Microscopic characterizations verify that only physical interactions occur during modification without generating new substances. The nano-filler facilitates the aggregation of small molecules and increases the proportion of macromolecules; meanwhile, it physically adsorbs light fractions and induces apparent redistribution of asphalt components, raising the relative proportion of resins and asphaltenes in the organic asphalt phase, realizing moderate heavy-fraction enrichment of the asphalt system. This study clarifies the internal correlation between molecular fraction evolution characteristics and macroscopic rheological performances of asphalt co-modified by nano-SiO_2_ and SBS, which can provide theoretical references for formula design and engineering application of modified asphalt materials.

## 1. Introduction

The SBS is the most widely used polymer modifier in pavement engineering. After being blended into asphalt, SBS can fully absorb light oil fractions to realize swelling and crosslinking, forming a continuous three-dimensional elastic network inside the asphalt matrix. This improves the elastic recovery capacity of asphalt and simultaneously enhances its basic high- and low-temperature performances, rendering it the mainstream technical solution for modified asphalt at present [1,2,3]. Nevertheless, abundant engineering practices and laboratory tests have verified that modification solely with single SBS still exhibits obvious performance deficiencies [4,5,6]. SBS-modified asphalt has limited reserve of high-temperature modulus; the elastic network is susceptible to slippage under high-temperature and heavy-load conditions, so its rutting resistance fails to satisfy the traffic demands of ultra-large axle loads [7,8]. Simply increasing the SBS dosage will significantly raise the construction viscosity of asphalt, increase the construction difficulty of mixing and paving, and substantially boost the overall material cost [9].

To remedy the deficiency of insufficient high-temperature stiffness of single-SBS-modified asphalt, inorganic nano-fillers have been gradually applied to the field of asphalt composite modification. Nano-silica (nano-SiO_2_) has attracted widespread attention in the industry owing to its merits of tiny particle size, large specific surface area and strong surface activity [10]. Existing studies demonstrate that uniformly dispersed nano-SiO_2_ inside the asphalt matrix can construct a micro rigid supporting skeleton, restrict the high-temperature flow of asphalt macromolecular chains, and effectively improve the complex modulus at high temperatures and resistance to permanent deformation of asphalt [11]. The hydrophilic surface of unmodified nano-SiO_2_ carries abundant silanol groups, which create poor interfacial affinity with nonpolar asphalt matrix and trigger severe particle agglomeration at high dosages. A recent comprehensive investigation compared hydrophilic and hydrophobic nano-silica in asphalt binders, confirming that surface hydrophobization drastically improves filler dispersion, interfacial compatibility and rheological performance by reducing surface polarity and weakening inter-particle hydrogen bonding forces [12]. The unmodified hydrophilic nano-SiO_2_ adopted in this study thus exhibits inherent compatibility limitations, which explains the gradual deterioration of performance when filler dosage exceeds the optimal threshold. Nevertheless, separate incorporation of inorganic nano-fillers brings prominent drawbacks. Inorganic particles possess poor compatibility with the organic asphalt matrix; excessive dosage easily triggers particle agglomeration and storage stratification of the system. Meanwhile, rigid nano-particles increase internal interfacial defects within asphalt, aggravate low-temperature brittleness, markedly reduce low-temperature stress relaxation capacity, and raise the risk of low-temperature cracking of pavements [13,14].

In view of the inherent drawbacks of the above single modifier, researchers have gradually carried out investigations on asphalt composite modified by polymers and inorganic nano-fillers. It is expected that the synergistic effect of the two modifiers can simultaneously and balanceably optimize the high- and low-temperature rheological performances of asphalt [15,16,17]. Existing studies have verified that the combined incorporation of SBS and inorganic nano-fillers can form a composite structure consisting of an organic elastic network and an inorganic rigid skeleton, thereby realizing bidirectional regulation to enhance high-temperature stiffness while retaining low-temperature toughness [18]. Nevertheless, current studies still have several obvious deficiencies, which restrict the standardized engineering application of composite modification technology in modified asphalt pavements. First of all, existing research on composite modified asphalt adopts relatively single performance evaluation methods, lacking a joint characterization system with multi-dimensional rheological indicators. Thus, it is difficult to fully reflect the viscoelastic evolution characteristics of asphalt over a wide temperature range [19,20]. Secondly, numerous studies only rely on conventional indicators such as penetration and softening point for analysis, with insufficient dimensions of rheological characterization. Such research cannot completely reveal the comprehensive rheological laws of modified asphalt regarding high-temperature deformation resistance, low-temperature crack resistance and anti-fatigue performance [21,22]. In terms of microscopic mechanism research, most existing studies on composite modified asphalt adopt morphological observation characterization methods, which can only intuitively observe the dispersion state of modified phases. It is hard to quantitatively reveal the internal modification mechanism from the perspectives of molecular scale and chemical fractions [23,24,25]. The combined application of multiple quantitative microscopic characterization methods can comprehensively analyze the microscopic evolution characteristics of modified systems from both molecular and fraction dimensions [26,27]. However, few relevant existing studies adopt a combined multi-method microscopic analysis scheme. The evolution mechanism of asphalt molecules and fractions under the synergistic effect of the two phases remains unclear, and sufficient experimental evidence is still absent for the quantitative structure–activity relationship between microscopic structures and macroscopic rheological performances [28,29]. In addition, systematic exploration on the optimal filler dosage range of this composite modification system has not yet been completed in existing research. There is a lack of systematic demonstration on the evolution laws of asphalt rheological performances and the stability characteristics of microscopic structures under different dosages, which fails to provide scientific references for the formula optimization of such modified asphalt [30,31].

To address the above research gaps and combine the actual service conditions of heavy-load asphalt pavements [32,33,34], virgin asphalt is selected as the research object in this study. A combined method of macroscopic performance tests and microstructure characterizations is adopted to systematically investigate the rheological evolution characteristics and microscopic synergistic modification mechanism of asphalt co-modified by nano-SiO_2_ and SBS. Conventional physical property tests of asphalt are carried out to characterize the variation in macroscopic performances of asphalt. Temperature sweep, MSCR, LAS and BBR tests are utilized to comprehensively evaluate the high- and low-temperature rheological performances, permanent deformation resistance and anti-fatigue performance of asphalt. FTIR, GPC and TLC-FID tests are integrated to reveal the evolution laws of functional groups, molecular sizes and chemical fractions of asphalt. The internal correlation between microstructure optimization and macroscopic rheological performance improvement of composite modified asphalt is clarified, and the synergistic modification mechanism of organic–inorganic two phases is elaborated. The research findings of this paper can enrich the theoretical system of asphalt composite modified by polymers and inorganic nano-fillers, and provide references for formula optimization, engineering application and performance improvement research of modified asphalt materials for heavy-traffic pavements.

## 2. Materials and Methods

### 2.1. Test Materials

#### 2.1.1. Virgin Asphalt

The 90# asphalt is selected as the virgin asphalt (VA) in this study. Basic performance tests are conducted in accordance with the specifications of JTG E20-2011 [35], and the test results are listed in Table 1.

#### 2.1.2. SBS Modifier

Linear SBS is adopted as the organic modifier in this study. This modifier possesses excellent compatibility with asphalt; upon heating, it rapidly swells and crosslinks to form a continuous elastic network within asphalt, which is widely utilized for preparing modified asphalt applied to heavy-load pavements. The modifier features stable physicochemical properties, and its fundamental technical parameters are presented in Table 2.

#### 2.1.3. Nano-SiO_2_

The Nano-SiO_2_ is selected as the inorganic modifying filler. This material features tiny particle size, large specific surface area and strong surface activity. Uniformly dispersed nano-SiO_2_ can construct a micro rigid supporting skeleton and enhance the high-temperature deformation resistance of asphalt. The nano-SiO_2_ adopted in this test possesses high purity and low particle agglomeration degree, and its main technical indexes are listed in Table 3.

### 2.2. Preparation of Composite Modified Asphalt Specimens

A unified standardized preparation procedure was adopted for all modified asphalt samples to guarantee the uniformity of each group and test repeatability. First, base asphalt was placed in a constant-temperature oven and heated to a molten flowable state under constant temperature for standby. The dosage of SBS was fixed uniformly; modifier particles were added into molten asphalt in batches. After preliminary manual stirring and mixing, constant-temperature shearing was performed under fixed temperature and shear rate to promote sufficient swelling and crosslinking of SBS, thereby preparing uniform and stable base SBS-modified asphalt. On this basis, multiple gradient dosages of nano-SiO_2_ were set. Nano-silica powder was incorporated into SBS-modified asphalt in batches, followed by continuous high-speed constant-temperature shearing to eliminate agglomeration and stratification of nano-particles. All blending and shearing processes were performed at a constant temperature of 170 °C with a high-speed shear rate of 5000 rpm. SBS swelling shearing lasted for 60 min, and subsequent nano-SiO_2_ dispersion shearing was maintained for 40 min to eliminate particle agglomeration. Post-shearing static maturation was conducted at 160 °C for 30 min before specimen sealing. After shearing, the specimens were kept at a constant temperature for static maturation. Finally, SBS/nano-SiO_2_ composite modified asphalt with different nano-filler dosages was obtained, sealed and stored at room temperature for all subsequent macroscopic performance tests and microscopic mechanism characterizations.

### 2.3. Test Methods

#### 2.3.1. Physical Property Test

Penetration, softening point and ductility tests of asphalt were strictly implemented in accordance with JTG E20-2011. A penetrometer was adopted for penetration measurement under the test temperature of 25 °C, applied load of 100 g and load holding time of 5 s. The ring-and-ball method was utilized for softening point testing with a heating rate of 5 °C/min. A ductilometer was used to conduct ductility tests at 5 °C with a stretching rate of 5 cm/min. Three parallel specimens were tested for each index, and the average value was taken as the final test result.

#### 2.3.2. Rheological Performance Tests

Temperature sweep test, MSCR test, LAS test and BBR test were adopted in this study to comprehensively evaluate the full-dimensional rheological performances of composite modified asphalt, including high-temperature stability, rutting resistance under heavy loads, medium-temperature anti-fatigue performance and low-temperature crack resistance. All rheological tests were strictly implemented in accordance with relevant test specifications.

(1) Temperature Sweep Test

The test temperature ranged from 46 °C to 88 °C with an interval of 6 °C, the loading frequency was set as 10 rad/s, the parallel plate gap was 1 mm, and the controlled strain was 0.5%. The complex shear modulus (G*) and phase angle (δ) of asphalt with different nano-filler dosages were recorded. The rutting factor (G*/sinδ) was calculated to evaluate the high-temperature viscoelasticity and shear resistance of asphalt.

(2) MSCR Test

The test temperature was fixed at 64 °C. Two stress levels of 0.1 kPa and 3.2 kPa were applied respectively, and 10 creep-recovery cycles were conducted at each stress level. Each cycle consisted of a 1 s creep stage and a 9 s recovery stage. The creep recovery rate (R) and non-recoverable creep compliance (Jnr) of asphalt were measured and calculated to characterize its permanent deformation resistance and high-temperature stability under repeated heavy loads.

(3) LAS Test

The test temperature was 25 °C with a loading frequency of 10 Hz. The strain amplitude increased linearly from 0.1% to 30%. The relationship between shear stress and strain of asphalt was recorded during testing. The fatigue life (N_f_) was calculated following the Viscoelastic Continuum Damage (VECD) framework specified in AASHTO TP 101-2012 [36]. A pre-test frequency sweep was first conducted to obtain linear viscoelastic parameters; damage characteristic curves were fitted to quantify internal damage accumulation, and failure criterion of 35% reduction in |G*|sinδ was adopted to determine the number of load cycles to N_f_ at target strain levels of 2.5%, 5% and 10%.

(4) BBR Test

The test temperatures were set as −12 °C, −18 °C and −24 °C, respectively. Asphalt specimens were fabricated into standard small beam samples, subjected to a constant load of 980 mN for 240 s. The creep stiffness (S) and creep rate (m) of asphalt at 240 s were recorded to evaluate the low-temperature stress relaxation and thermal shrinkage crack resistance of asphalt. Smaller S and larger m values indicate better low-temperature toughness of asphalt.

#### 2.3.3. Microstructure Characterization Tests

Three kinds of microscopic characterization tests including FTIR, GPC and TLC-FID were carried out to reveal the evolution law of asphalt microstructure under the synergistic modification of SBS and nano-SiO_2_ from multi-scales of functional group structure, molecular size and chemical components, and clarify the internal microscopic mechanism for the improvement of macroscopic rheological properties.

(1) FTIR Test

A Fourier transform infrared spectrometer was adopted, and asphalt samples were prepared by the KBr tablet pressing method. The test wavenumber range was 4000~400 cm^−1^, the spectral resolution was set to 4 cm^−1^. The changes in position, peak intensity and peak area of characteristic infrared spectrum peaks of base asphalt, SBS-modified asphalt and SBS/nano-SiO_2_ composite modified asphalt were compared to judge whether physical blending was the main interaction among raw materials in the modified system or wheter chemical reactions existed, and to reveal the type of modification effect from the perspective of functional groups.

(2) GPC Test

A gel permeation chromatograph was used with tetrahydrofuran as the mobile phase at a flow rate of 1 mL/min and a test temperature of 35 °C. Asphalt samples were dissolved in tetrahydrofuran to prepare a solution with a concentration of 2 mg/mL, and the solution was filtered through a filter membrane before testing. The asphalt-THF solution was gently filtered through a 0.45 μm PTFE membrane to remove large insoluble nano-SiO_2_ agglomerates only; insoluble high-molecular-weight asphaltene aggregates were recovered from filter residues, redissolved and incorporated into molecular weight calculation to fully characterize the complete molecular distribution of the organic asphalt phase.

(3) TLC-FID Test

A combined device of thin-layer chromatograph and flame ionization detector was adopted. Asphalt samples were dissolved in trichloromethane and spotted on silica thin-layer rods. N-heptane, toluene, and a mixed solution of toluene and ethanol were used for segmented elution in sequence to separate four major fractions of asphalt, namely saturates, aromatics, resins and asphaltenes. The content of each fraction was quantified by the FID detector. All SARA fraction mass fractions reported in this study were normalized to 100% of the extractable organic asphalt phase, with inorganic nano-SiO_2_ fillers excluded from quantitative calculation to eliminate dilution effects caused by solid filler addition.

### 2.4. Test Condition Design

To explore the influence law of different nano-silica dosages on the macroscopic pavement performance and microscopic modification mechanism of SBS composite modified asphalt, a single-variable experimental design method was adopted in this study. The dosage of SBS modifier was fixed, and composite modified asphalt was prepared with multiple gradient dosages of nano-SiO_2_. The sample preparation procedure, test environment and test parameters were kept consistent for all specimens; only the dosage of inorganic nano-filler was adjusted. This design effectively avoids experimental errors and ensures favorable comparability and regularity of test results.

A total of seven groups of asphalt specimens were set in this research, including a blank group of base asphalt, a control group of single-SBS-modified asphalt, and five experimental groups of composite modified asphalt with different nano-SiO_2_ dosages. All dosages were calculated as mass fractions based on the mass of base asphalt. The serial numbers and specific mixing proportions of each specimen group are listed in Table 4.

## 3. Results and Discussion

### 3.1. Conventional Physical Properties

**Figure 1:**
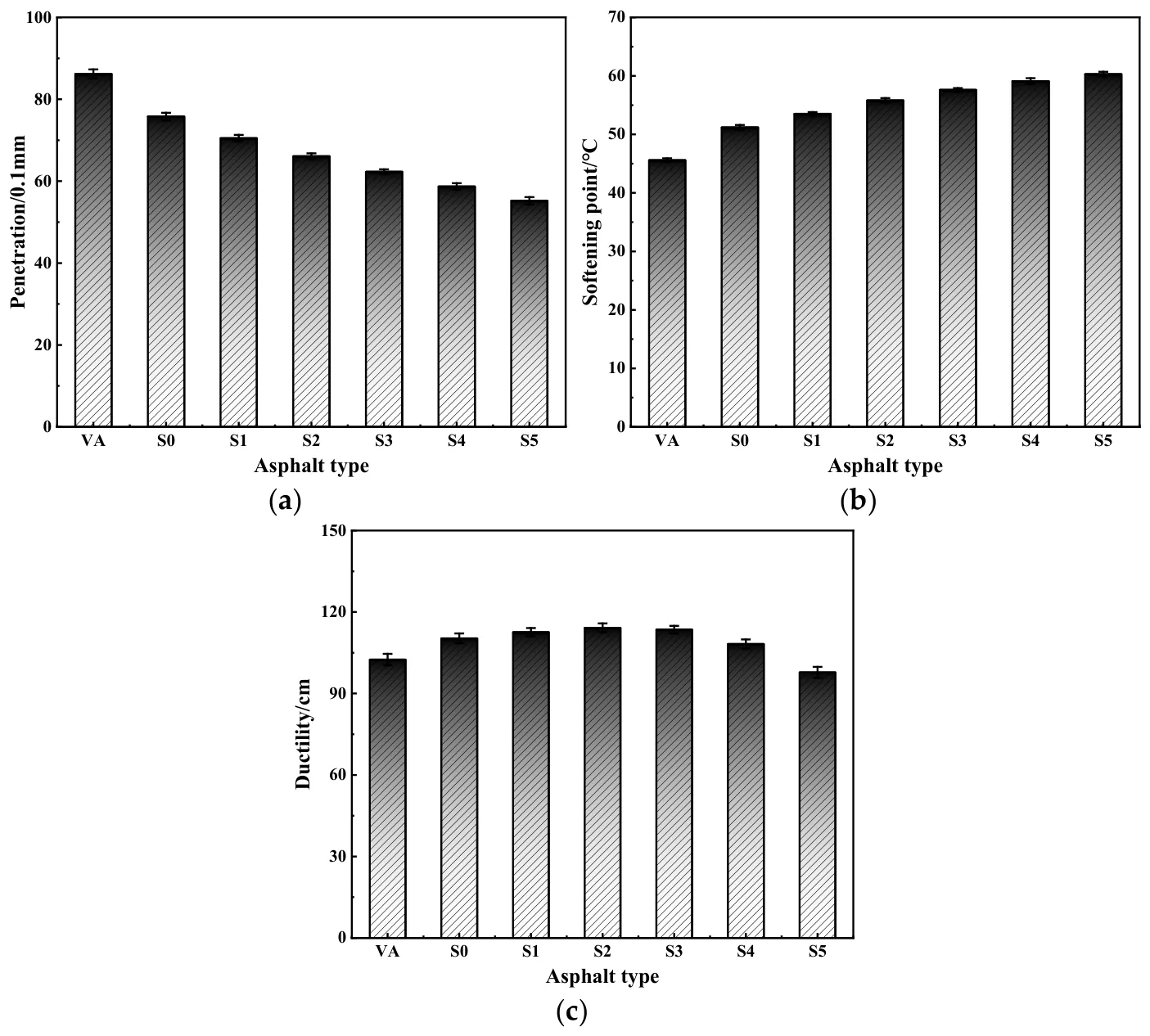
Conventional physical properties of different asphalts: (**a**) penetration; (**b**) softening point; (**c**) ductility.

The penetration, softening point and ductility are core indicators characterizing the macroscopic physical performances of asphalt, which can directly reflect the hardness, flowability and plasticity of asphalt. Standardized unified tests were carried out on seven groups of asphalt specimens labeled VA, S0~S5 in this study, and the detailed test results are displayed in Figure 1.

It can be seen from Figure 1 that the VA sample presents a relatively high penetration value and low softening point. Under high-temperature conditions, internal asphalt molecules are prone to slippage and flow, leading to weak resistance to softening and deformation. Meanwhile, its low-temperature ductility is limited with insufficient deformation allowance at low temperatures, so cracking damage easily occurs under temperature variation. For sample S0 blended with a fixed dosage of modifier, all three physical indicators are comprehensively improved: penetration decreases obviously, while softening point and low-temperature ductility increase simultaneously. Such variations originate from the swelling and crosslinking of polymer chain segments inside molten asphalt, which constructs a continuous elastic network. This network restricts the free flow of light fractions within the system. The abundant polar silanol groups distributed on the hydrophilic nano-SiO_2_ surface form hydrogen bonds and van der Waals interactions with polar components in asphalt, enabling selective physical immobilization of low-polarity saturates and aromatics. As reported in [24], surface hydrophobization of silica can adjust the strength of component adsorption, balance the rigidity of inorganic skeleton and low-temperature flexibility of asphalt, and further optimize the crosslinked composite network constructed by SBS elastic chains and nano-rigid particles. In addition, flexible polymer chains enhance the low-temperature deformability of asphalt, which represents the typical performance evolution rule caused by modification treatment. When nano-SiO_2_ is gradually added based on SBS-modified asphalt S0, penetration and softening point show a monotonic variation trend.

As the filler dosage increases continuously, penetration decreases steadily while the softening point rises synchronously and gradually. For medium and low dosage groups including S1, S2 and S3, nano-SiO_2_ particles can evenly fill the internal pores of the elastic network. Relying on their large specific surface area, these particles adsorb light fractions such as saturates and aromatics inside asphalt, building an inorganic rigid supporting skeleton within the system. This skeleton further restricts the slippage of asphalt molecules under high-temperature conditions, thereby continuously improving the room-temperature hardness and high-temperature thermal stability of asphalt. Even when the filler dosage is increased to the range of S4 and S5, penetration still slightly declines and the softening point slightly rises, which indicates that the effect of nano-SiO_2_ on improving the high-temperature stiffness of asphalt will not disappear completely due to particle agglomeration. Low-temperature ductility presents a unimodal trend of first increasing and then decreasing with the rising dosage of nano-SiO_2_, and the inflection point with optimal performance appears in group S2. In the dosage range from S0 to S2, filler particles disperse uniformly without destroying the continuous elastic phase inside the system. The uniformity of asphalt colloid is improved, low-temperature plasticity increases moderately, and ductility reaches the peak value among all specimens. When the filler dosage exceeds 2%, the agglomeration of nano-particles becomes increasingly prominent. Agglomerate masses split the continuous elastic phase and generate numerous weak interfacial defects inside the system. Under low-temperature loads, stress concentrates at these defects, resulting in continuous attenuation of asphalt plasticity. The ductility value of S5 is even lower than that of S0.

Horizontal comparison of data from all specimens demonstrates that nano-SiO_2_ and SBS modifier exert a synergistic modification effect. The dosage range of 1% to 3% can simultaneously optimize the high-temperature stability and low-temperature plasticity of asphalt. When the dosage exceeds 3%, the adverse effect of deteriorated low-temperature performance gradually becomes dominant. Considering the overall performance of the three indicators, the optimal dosage of nano-SiO_2_ in this experimental system is 2%.

### 3.2. Analysis of Rheological Properties

#### 3.2.1. Temperature Sweep Test

**Figure 2:**
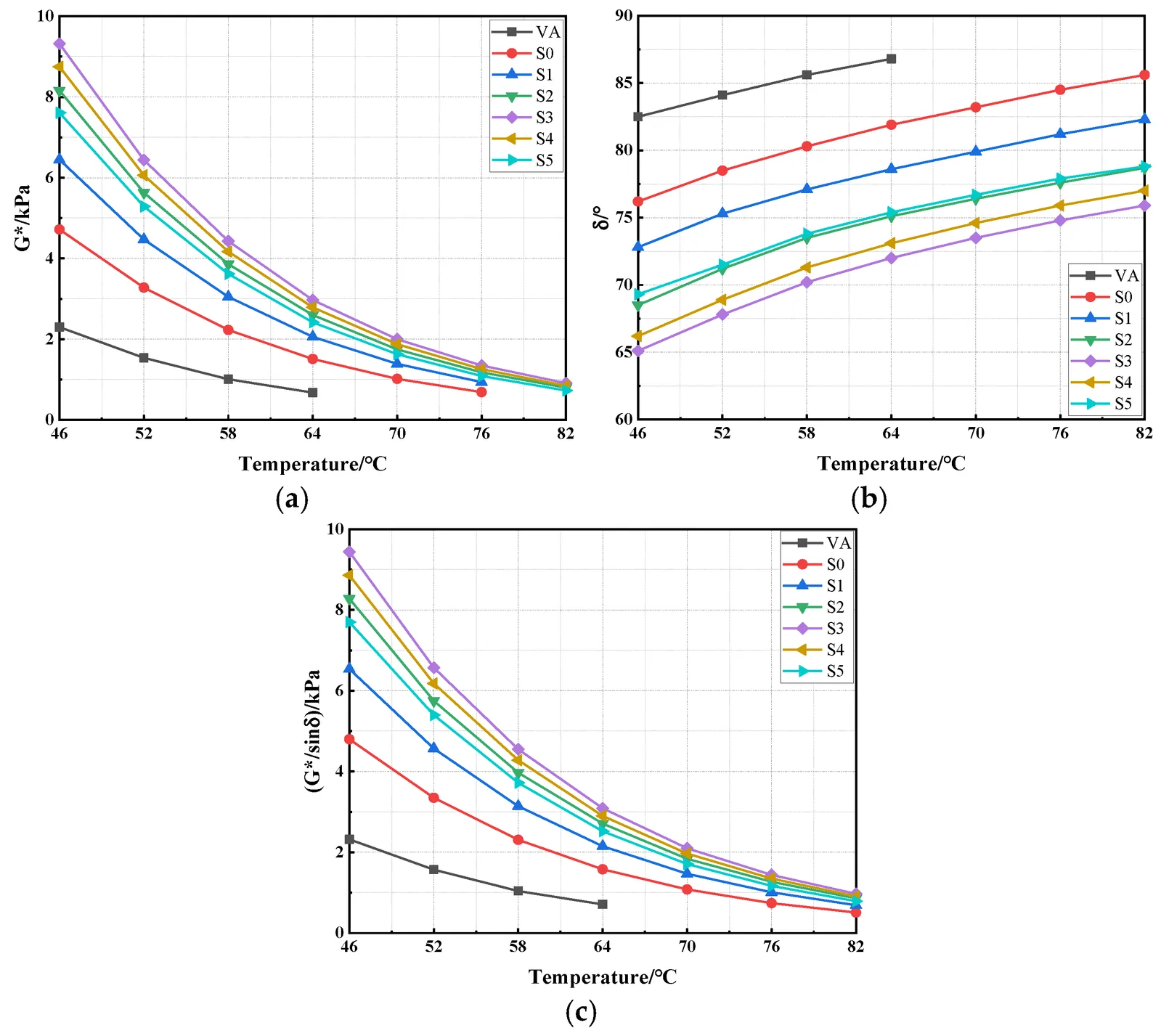
The high-temperature performance of several asphalts: (**a**) G*; (**b**) δ; (**c**) G*/sinδ.

The G*, δ and G*/sinδ are core rheological parameters for characterizing the high-temperature viscoelastic features of asphalt, which can accurately reflect the high-temperature stiffness, proportion of elastic components and shear deformation resistance of asphalt. In this study, wide-range temperature sweep tests from 46 °C to 88 °C were conducted on all specimens including VA and S0 to S5, and the test results are shown in Figure 2.

It can be observed from Figure 2 that within the entire test temperature range, VA exhibits the lowest G* value and the largest δ value among all specimens, showing obvious characteristics of viscous fluid. Internal asphalt molecules are prone to relative slippage under high-temperature loads, leading to poor resistance to high-temperature deformation. After S0 is prepared by adding the modifier at a fixed dosage, a continuous elastic network forms inside asphalt, and the proportion of elastic components in the system increases substantially. This is directly reflected in the overall rise in G*, synchronous decline of δ and remarkable growth of G*/sinδ, which delivers a basic improvement in the high-temperature shear deformation resistance of asphalt.

After nano-SiO_2_ was added in gradient dosages based on S0, the G*/sinδ of composite modified asphalt achieved a secondary increase. The growth range of performance rose first and then weakened with the increase in filler dosage. Inside medium and low dosage samples S1, S2 and S3, uniformly dispersed nano-SiO_2_ particles fill the internal pores of the elastic network, increasing the intermolecular friction of asphalt and the overall structural rigidity, which effectively restricts the slippage of asphalt molecules under high-temperature conditions. When the filler dosage was increased to high-dosage groups S4 and S5, the agglomeration of nano-particles became obvious, and multiple stress concentration regions formed inside the system. Accordingly, the modification benefit brought by nano-filler gradually declined.

As the test temperature rises continuously, the G* of all specimens decreases synchronously, while δ keeps increasing. High-temperature gradually relaxes the internal elastic network of asphalt, and the characteristics of viscous fluid gradually become dominant. At the same test temperature, the G*/sinδ values of all composite modified asphalt samples S1 to S5 are always higher than those of S0 and VA. This directly reflects the synergistic effect formed by the elastic network and rigid skeleton of nano-SiO_2_, which can durably improve the high-temperature rheological stability of asphalt.

#### 3.2.2. MSCR Test

**Figure 3:**
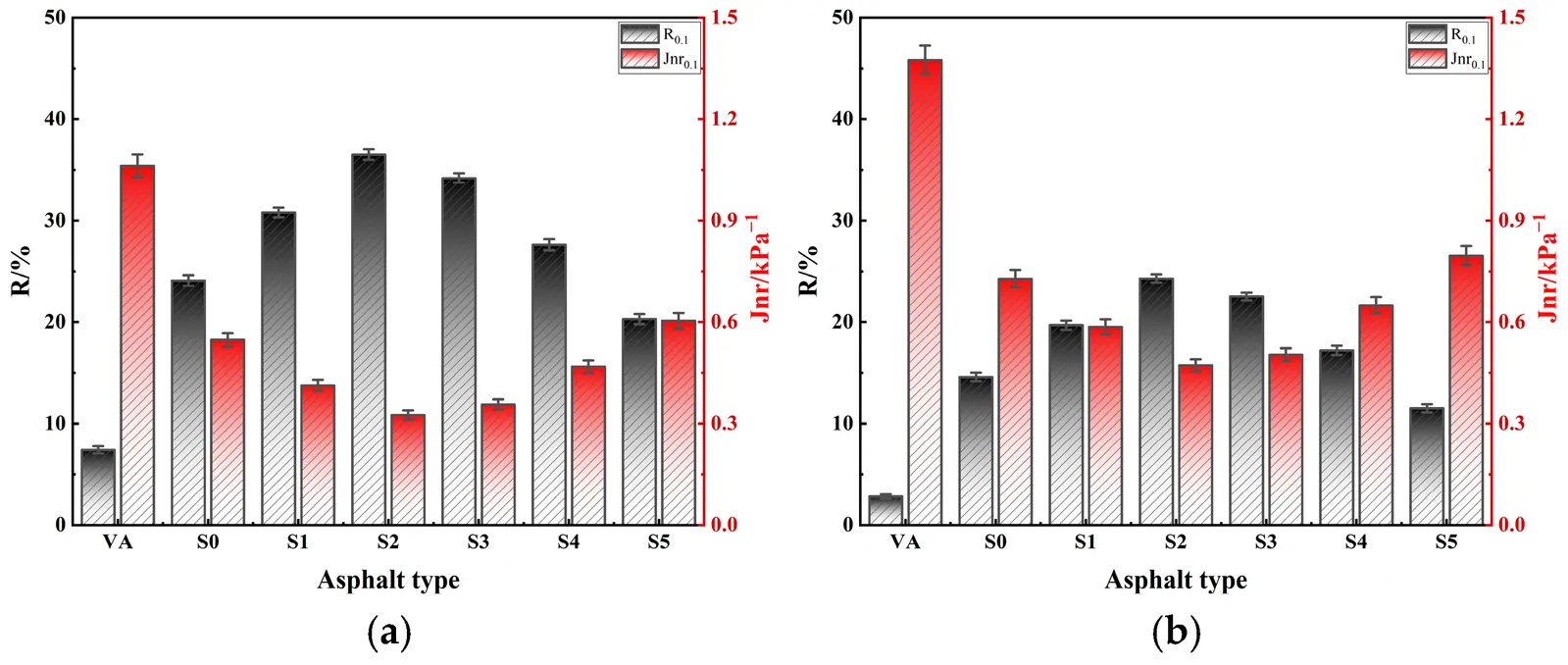
High-temperature deformation resistance of different asphalts: (**a**) 0.1 kPa; (**b**) 3.2 kPa.

The MSCR test is adopted to evaluate the creep deformation and elastic recovery characteristics of asphalt under cyclic high-temperature loads, which can accurately reflect its permanent deformation resistance and high-temperature rutting resistance potential under actual service conditions. The unified test temperature was set at 64 °C, and the test results are shown in Figure 3.

It can be seen from Figure 3 that VA has the minimum R value and maximum Jnr value. Without the support of a continuous internal elastic network, the proportion of plastic deformation rebound under load is very low, so irreversible ruts are easily formed on pavements under high-temperature. After adding modifier to prepare S0, a cross-linked elastic network forms inside asphalt, and its recovery capacity against shear deformation is significantly improved. R rises and Jnr declines under both stress levels, achieving a basic improvement in high-temperature deformation resistance.

When nano-SiO_2_ is added in gradient dosages based on S0, the rebound performance shows a trend of first increasing and then decreasing. In medium and low dosage groups S1, S2 and S3, uniformly dispersed nano-particles fill the gaps of the elastic network to form a rigid supporting structure, which effectively restrains the irreversible flow of asphalt. R gradually increases and Jnr gradually decreases under two stress levels, reaching the optimal state at S2. When the filler dosage is increased to S4 and S5, nano-particles agglomerate, split the continuous elastic phase and generate abundant interfacial defects inside the system. Obvious stress concentration occurs under load, the deformation recovery capacity decreases, and the proportion of permanent deformation rises.

Comparison of data under two loading levels reveals that all samples show decreased R and increased Jnr during the high-stress stage from 100 s to 200 s. Heavy-load shear damages the elastic structure of asphalt and accelerates the accumulation of permanent deformation. The composite modified asphalt S1 to S3 presents smaller differences in indicators between low and high stress levels. The elastic network and nano rigid skeleton produce synergistic effects, leading to more prominent anti-deformation advantages under heavy load conditions. Considering all test results under two stress levels, S2 possesses the best comprehensive high-temperature rutting resistance performance.

#### 3.2.3. LAS Test

**Figure 4:**
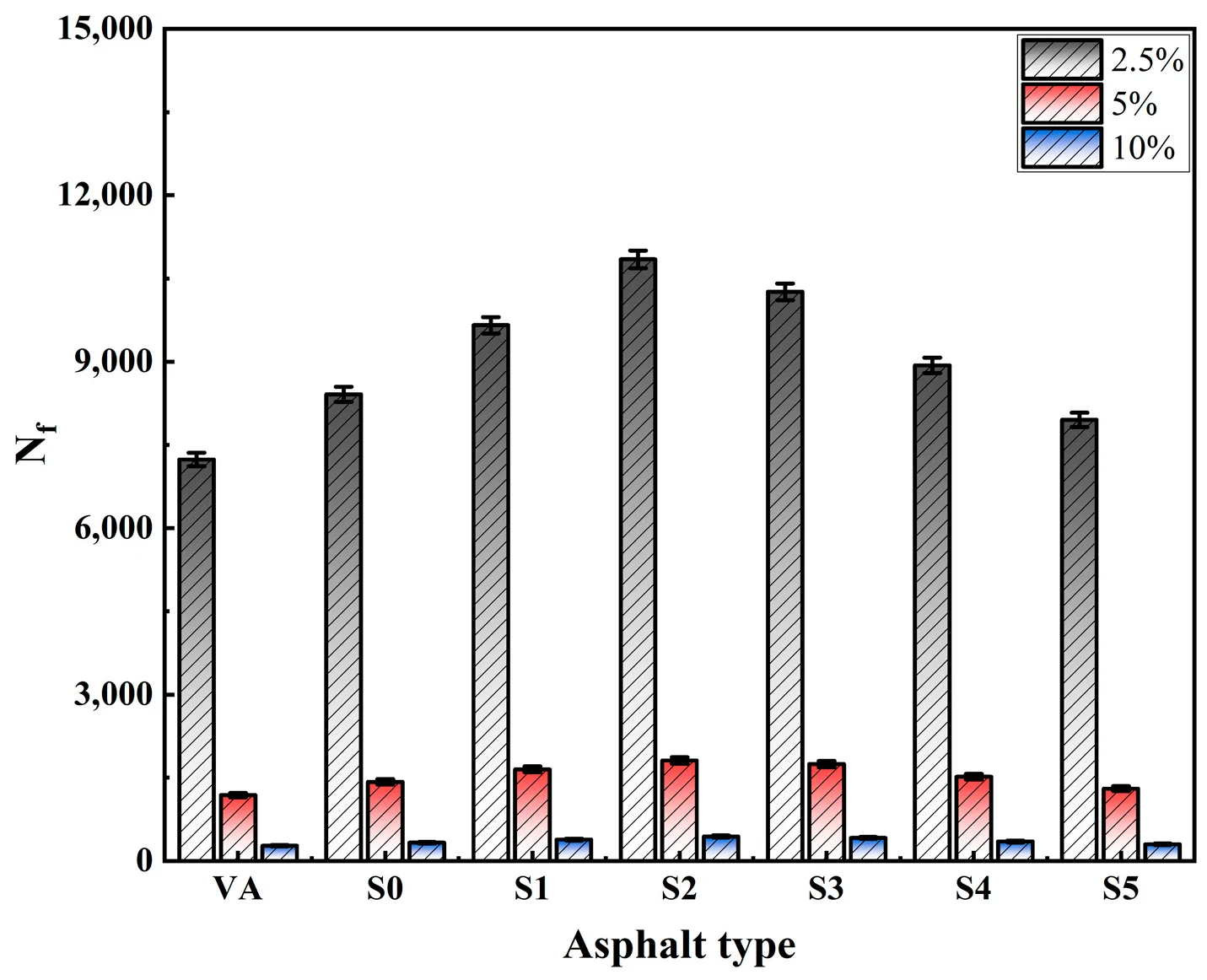
Fatigue performance of different asphalts.

The N_f_ is the core parameter to characterize the fatigue durability of asphalt under cyclic loads, which can quantitatively reflect the evolution rate of internal damage under different strain levels. To evaluate the fatigue performance of various asphalt binders, three typical strain levels of 2.5%, 5% and 10% were selected to carry out LAS tests on all asphalt specimens, and the test results are shown in Figure 4.

It can be seen from Figure 4 that for the same specimen, the larger the applied strain amplitude, the lower the N_f_ value. High strain continuously stretches the asphalt colloidal structure, accelerates the generation and propagation of microcracks, and greatly raises the accumulation rate of fatigue damage. Horizontal comparison of each group shows that VA lacks a cross-linked elastic network, so stress can hardly be dispersed under cyclic loads, resulting in the lowest N_f_ values under all three strain levels. After adding modifier to prepare S0, a continuous elastic network forms in the system. This network can buffer cyclic stress and delay the initiation of microcracks, which significantly increases N_f_ under each strain level and optimizes the basic anti-fatigue performance of asphalt.

When nano-SiO_2_ is added in gradient dosages based on S0, N_f_ presents a trend of first increasing and then decreasing. Nano-particles disperse uniformly in S1 and S2, which can disperse local stress concentration zones and reduce crack initiation sites, slowing down damage development. N_f_ rises gradually and reaches its peak at S2; the performance of S3 declines slightly. When the dosage is increased to S4 and S5, nano-particles agglomerate and form numerous weak interfaces inside the system. Cracks easily expand rapidly along these interfaces under cyclic loads, leading to continuous deterioration of fatigue performance. The agglomeration threshold is governed by the balance between filler-asphalt interfacial affinity and inter-particle surface attraction. At dosages exceeding 2%, the total surface area of nano-SiO_2_ exceeds the adsorption capacity of asphalt maltenes; insufficient organic coating on particle surfaces strengthens inter-particle hydrogen bonding between exposed silanol groups, leading to spontaneous stacking and agglomeration. Such structural evolution of nano-particles networks at high filler loadings and corresponding rheological deterioration have been systematically illustrated in previous nano-silica-asphalt research [34].

Comparison of the three strain conditions reveals obvious performance gaps between groups under the low strain of 2.5%. Under the high strain of 10%, both the elastic network and nano supporting skeleton suffer severe shear damage, and the differences in N_f_ values among different specimens are obviously narrowed. Based on all test data under various strains, S2 has excellent rutting resistance and fatigue resistance, representing the optimal mixing proportion in this experiment.

#### 3.2.4. BBR Test

**Figure 5:**
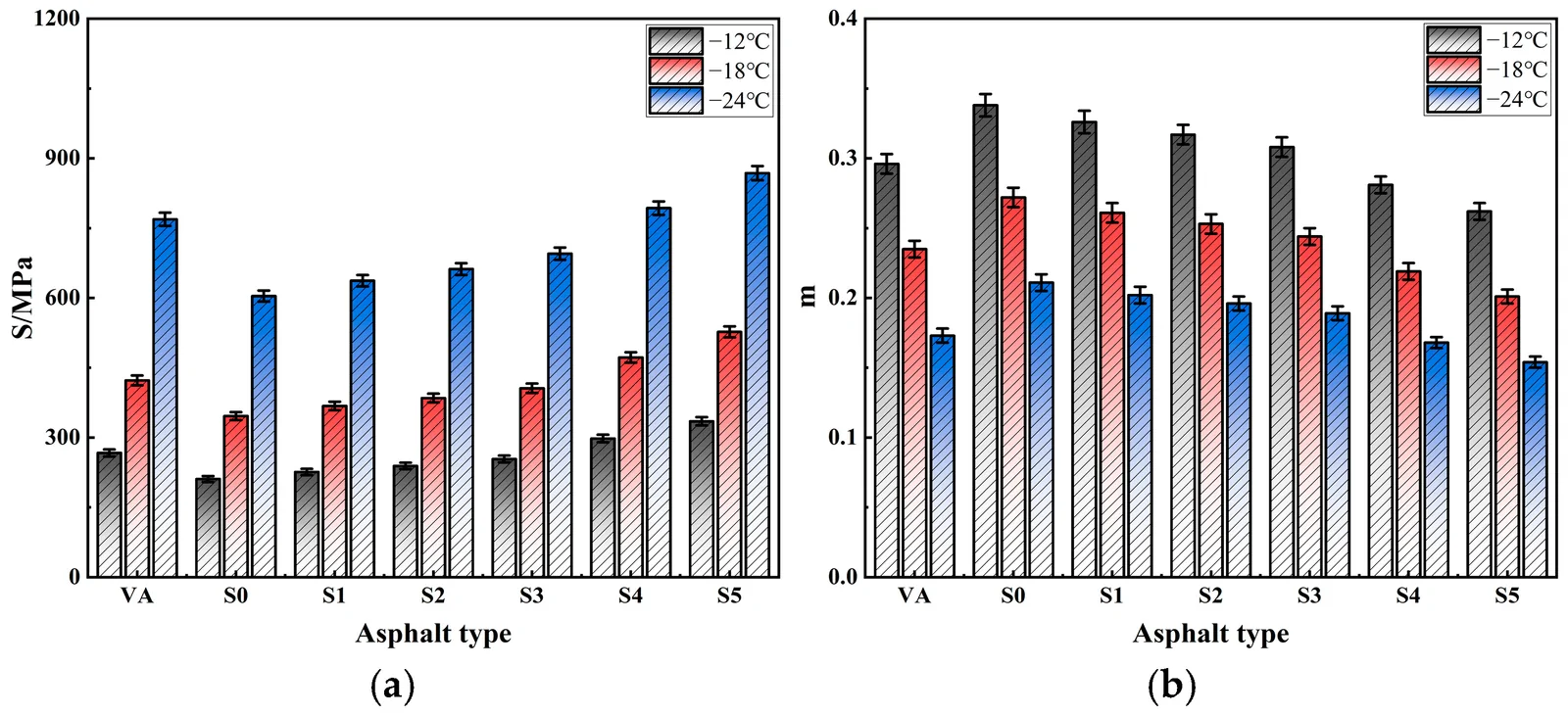
Low-temperature performance of different asphalt binders: (**a**) S; (**b**) m.

The BBR test is used to evaluate the low-temperature bending creep property and crack resistance potential of asphalt. By measuring S and m, the influence law of multi-coupled conditions on the low-temperature viscoelasticity and thermal stress relaxation capacity of asphalt can be quantitatively revealed. Three standard low temperatures of −12 °C, −18 °C and −24 °C were set for the test, with S and m as core evaluation indicators. The BBR test results are shown in Figure 5.

It can be observed from Figure 5 that low-temperature restricts the movement of asphalt molecular chain segments and causes overall hardening of the colloid. No modified components are added to VA, and there is no flexible polymer chain buffer structure inside. Under all three test temperatures, VA shows the maximum S value and minimum m value among all specimens. Internal stress generated by low-temperature shrinkage cannot be released in a timely manner, so thermal shrinkage cracks are easily formed on pavements at low temperatures. After S0 is prepared by adding modifier at a fixed dosage, flexible elastic chains are introduced into the system, and the low-temperature deformation allowance of asphalt is improved. Under identical low-temperature conditions, S decreases obviously while m rises synchronously, achieving an improvement in basic low-temperature crack resistance.

After nano-SiO_2_ is added in gradient dosages based on S0, the low-temperature indicators change regularly. For groups S1, S2 and S3, creep stiffness S moderately increases while m slightly declines, indicating slightly weakened low-temperature stress relaxation capacity relative to S0. Nevertheless, all composite modified specimens still exhibit markedly superior low-temperature cracking resistance compared with VA, as their S values remain much lower and m values much higher than those of VA under identical test temperatures. Rigid inorganic fillers synergistically enhance high-temperature performance with only a slight adverse impact on low-temperature stress relaxation capacity. When the filler dosage is increased to high-dosage groups S4 and S5, massive nano agglomerates form and the number of interfacial defects inside the system rises sharply. Low-temperature stress tends to concentrate at these defects, resulting in a sharp increase in S and obvious reduction in m, accompanied by significant deterioration of low-temperature crack resistance.

Vertical comparison of the three test temperatures indicates that lower temperature leads to synchronous growth of S and continuous decline of m for all specimens, and the low-temperature hardening effect becomes increasingly severe. Based on all test data under various temperatures, S2 balances multiple performances including high-temperature rutting resistance, fatigue resistance and low-temperature crack resistance, and possesses the optimal comprehensive pavement performance balance.

### 3.3. Micro-Composition and Structure Characterization of Modified Asphalt

To reveal the internal chemical mechanism accounting for the differences in macroscopic rheological properties of nano-SiO_2_ composite modified asphalt, three microscopic test methods including FTIR, GPC and TLC-FID were adopted for comparative analysis of all specimens (VA and S0~S5) from three dimensions: functional group structure, molecular weight distribution and four-component composition of asphalt.

#### 3.3.1. FTIR Test

**Figure 6:**
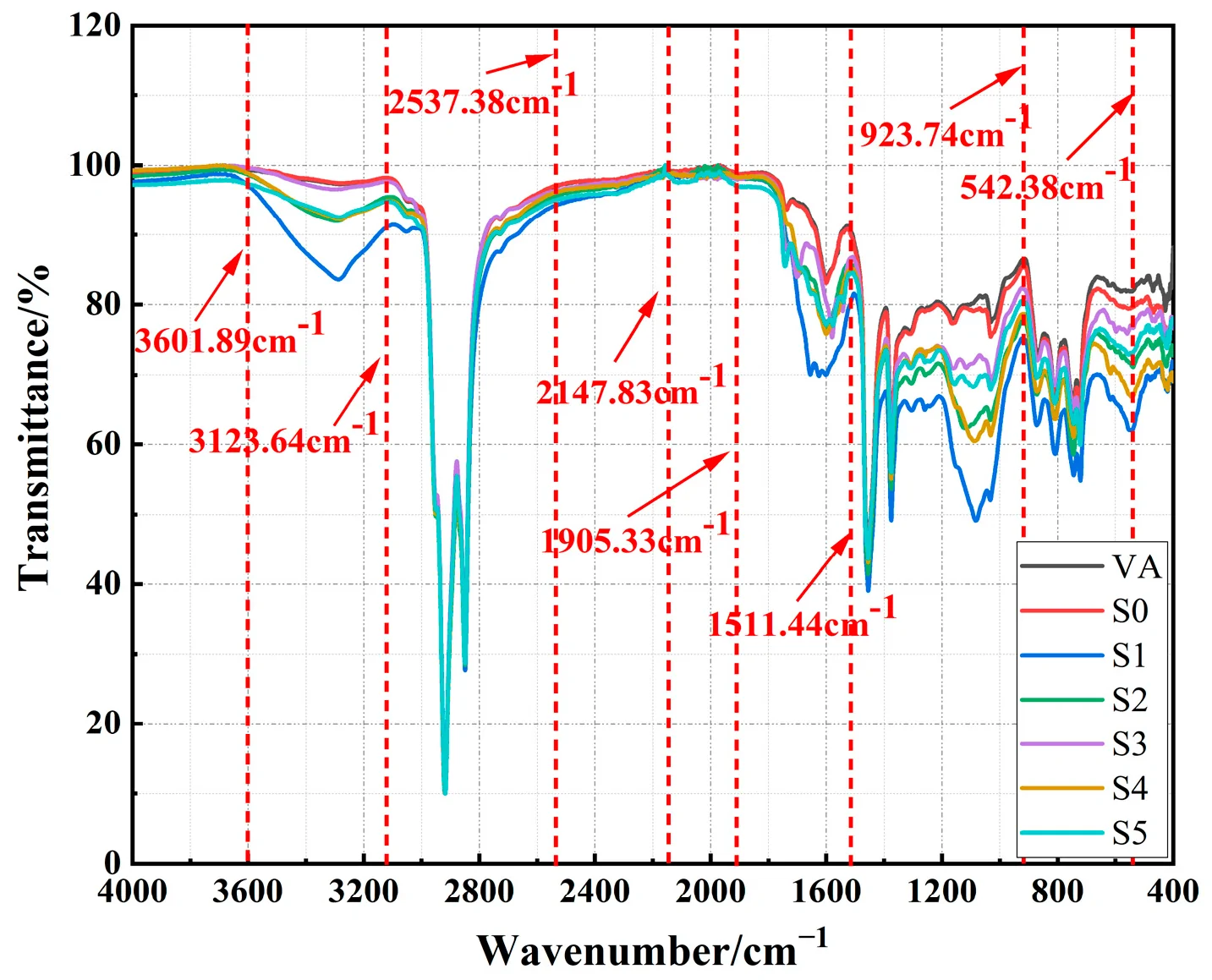
Microstructure of different asphalts.

The FTIR test can qualitatively identify the type of interfacial interaction among base asphalt, polymer modifier and nano-SiO_2_ through the variations in position, intensity and shape of characteristic absorption peaks, distinguish chemical crosslinking from physical adsorption, and clarify the internal binding mechanism of the composite system. Therefore, FTIR tests were carried out on all asphalt specimens in this study, and the test results are presented in Figure 6.

It can be seen from Figure 6 that only absorption bands corresponding to native functional groups of asphalt exist in the spectrum of VA. The region of 3123.64~3601.89 cm^−1^ belongs to hydroxyl stretching vibration with weak absorption intensity; the region of 542.88~923.74 cm^−1^ is the characteristic zone of Si-O-Si without obvious characteristic peaks. This phenomenon indicates that VA contains a low content of polar groups, and molecules are only bound by weak van der Waals forces, which cannot effectively restrict light fractions in the system.

After preparing S0, no new characteristic absorption bands appear across the full wavenumber range, and only the absorption intensity of original bands changes. The hydroxyl stretching vibration zone at 3123.64~3601.89 cm^−1^ shows remarkably enhanced absorption intensity compared with VA; the C=C skeleton stretching vibration at 1511.44 cm^−1^ also presents synchronously increased absorption intensity. The above variations demonstrate that modifiers only intertwine and embed with VA through hydrogen bonds and polar molecular forces without the generation of covalent bonds, and merely physical blending occurs in the system. The continuous elastic network formed by molecular entanglement can bind light fractions, disperse external shear stress and reduce the possibility of colloidal slip failure, so all rheological indicators of S0 outperform VA. S1 to S5 are prepared sequentially based on S0. Distinct characteristic peaks emerge in the Si-O-Si characteristic zone of 542.88~923.74 cm^−1^ starting from S1, and the absorption intensity keeps rising with the increase in filler dosage. The peak shapes of corresponding bands for S1, S2 and S3 are narrow and symmetrical, which means nano-particles can evenly fill the gaps of elastic networks. Silanol groups on particle surfaces adsorb saturates and aromatics inside the system to further stabilize the colloidal structure. Among all groups, S2 achieves the optimal particle dispersion state and the best comprehensive macroscopic performance. The corresponding bands of S4 and S5 exhibit obvious broadening, tailing and distortion, which is caused by massive agglomeration and stacking of nano-particles. The agglomerates split the original continuous composite phase and generate numerous weak stress interfaces inside the system.

Based on all FTIR test results, no characteristic absorption bands corresponding to chemical reactions are observed in the spectra of VA, S0 and S1~S5. The modification effect of the whole system only relies on physical adsorption and physical molecular entanglement, without chemical crosslinking reactions. An appropriate amount of filler can form a synergistic effect with the internal elastic network of S0 and optimize the microscopic colloidal structure; excessive filler dosage triggers particle agglomeration and destroys colloidal continuity. The physical adsorption and intermolecular entanglement confirmed by FTIR provide the fundamental precondition for the improved rheological performance of composite modified asphalt. Without chemical crosslinking, the synergistic organic-inorganic network can stably increase complex modulus and elastic recovery capacity under high-temperature repeated loads, which well explains the optimal rutting resistance and fatigue durability observed in temperature sweep, MSCR and LAS tests of the 2% nano-SiO_2_ group.

#### 3.3.2. GPC Test

**Figure 7:**
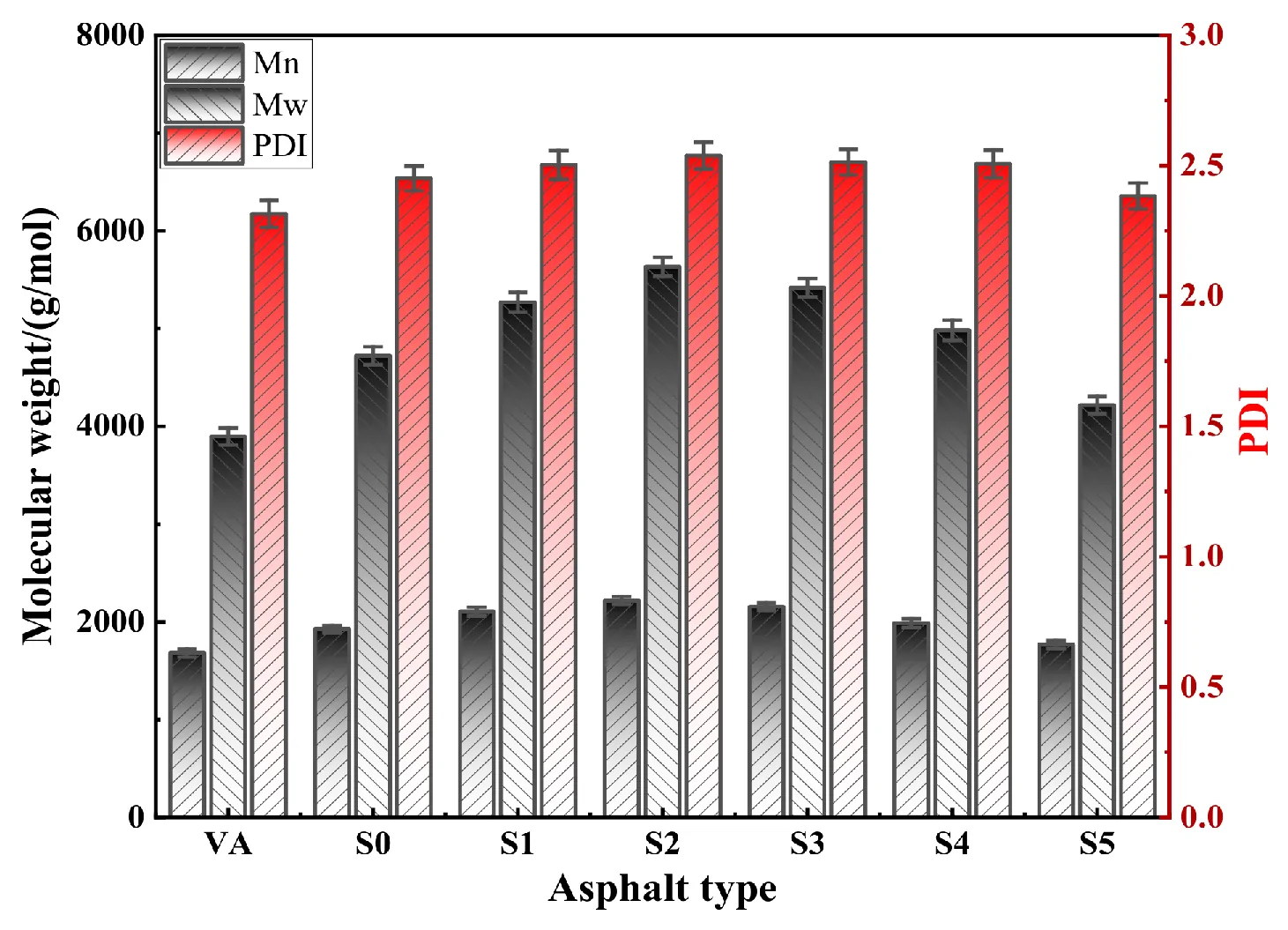
Variation law of molecular weight of different asphalts.

The GPC characterizes the variation rules of molecular weight and molecular weight distribution index inside VA, S0 and S1~S5 according to the distribution features of elution curves corresponding to different retention times, and reveals the regulation mechanism of modification on the proportion of macromolecular chain segments and light small-molecule fractions in the system. To evaluate the molecular weight variations in different asphalt binders, GPC tests were carried out on all specimens, and characteristic parameters including Mn, Mw and PDI were calculated. The test results are shown in Figure 7.

It can be seen from Figure 7 that Mn and Mw show an overall trend of continuous increase at first, followed by gradual decline after reaching the peak value. VA possesses the minimum Mn and Mw among all specimens. After preparing S0, both parameters rise synchronously, increasing by 14.5% and 21.2% respectively compared with VA. When the filler is further added to prepare S1 and S2, the molecular weight keeps increasing and reaches the peak value of all groups at S2. Starting from S3, the two parameters decline slightly, and the decreasing range continues to expand for S4 and S5. At S5, the molecular weight is only slightly higher than VA by 5.1% and 8.2%, indicating a sharp shrinkage of the overall molecular size. This variation trend reflects that after the modifier is incorporated, it can intertwine and associate with small asphalt molecules to form abundant composite macromolecular aggregates, which directly raise the overall average molecular size of the system. With the gradual addition of fillers, active sites on filler surfaces can further adsorb light small-molecule fractions of asphalt and promote the generation of more macromolecular aggregates, leading to continuous growth of molecular size. When the filler dosage exceeds a reasonable range, particle agglomeration becomes dominant. Agglomerates cannot be uniformly embedded into the molecular chain network; instead, they split the formed macromolecular association structure, part of the adsorbed small molecules dissociate again, and the average molecular weight of the system decreases continuously accordingly.

In terms of the variation trend of PDI, its overall changing tendency is consistent with Mn and Mw, also presenting a unimodal characteristic of rising first and then falling. VA has the lowest PDI of all groups. After the modifier is introduced to prepare S0, PDI rises slightly, continues to increase for S1 and S2 and reaches the maximum value at S2. The PDI of S3 and S4 drops slightly and remains within a similar range, while S5 shows an obvious decline, and the overall uniformity of molecular distribution returns close to that of VA. PDI represents the width of molecular weight distribution; a higher value indicates a higher coexistence degree of macromolecular and small-molecule fractions in the system. The continuous growth of PDI from S0 to S2 is attributed to the synergistic effect of modifiers and fillers continuously generating macromolecular aggregates, increasing the gradient of molecular sizes in the system and broadening the distribution range. After S2, filler agglomeration destroys the stable molecular association structure, macromolecular aggregates dissociate, the range of molecular sizes narrows, and thus PDI gradually decreases.

Based on the variation rules of molecular weight parameters, modifiers can effectively increase the average molecular size of asphalt and broaden molecular weight distribution. Matching with filler at an appropriate dosage can further amplify this effect. Under the mixing proportion of S2, the synergistic effect of molecular entanglement and filler adsorption is the strongest, the quantity of macromolecular aggregates is the largest, and the molecular distribution structure is optimal. Excessive filler dosage leads to particle agglomeration, damages the stable macromolecular network, reduces the average molecular size and narrows molecular weight distribution, which greatly weakens the modification effect at the molecular aggregation level. The increased macromolecular proportion and wider molecular weight distribution at 2% nano-SiO_2_ directly restrict the sliding of asphalt molecular chains under thermal and shear loads. This molecular aggregation effect raises the rutting factor at high-temperature and slows microcrack propagation under cyclic strain, consistent with the superior high-temperature stiffness and fatigue life obtained from rheological tests.

#### 3.3.3. TLC-FID Test

**Figure 8:**
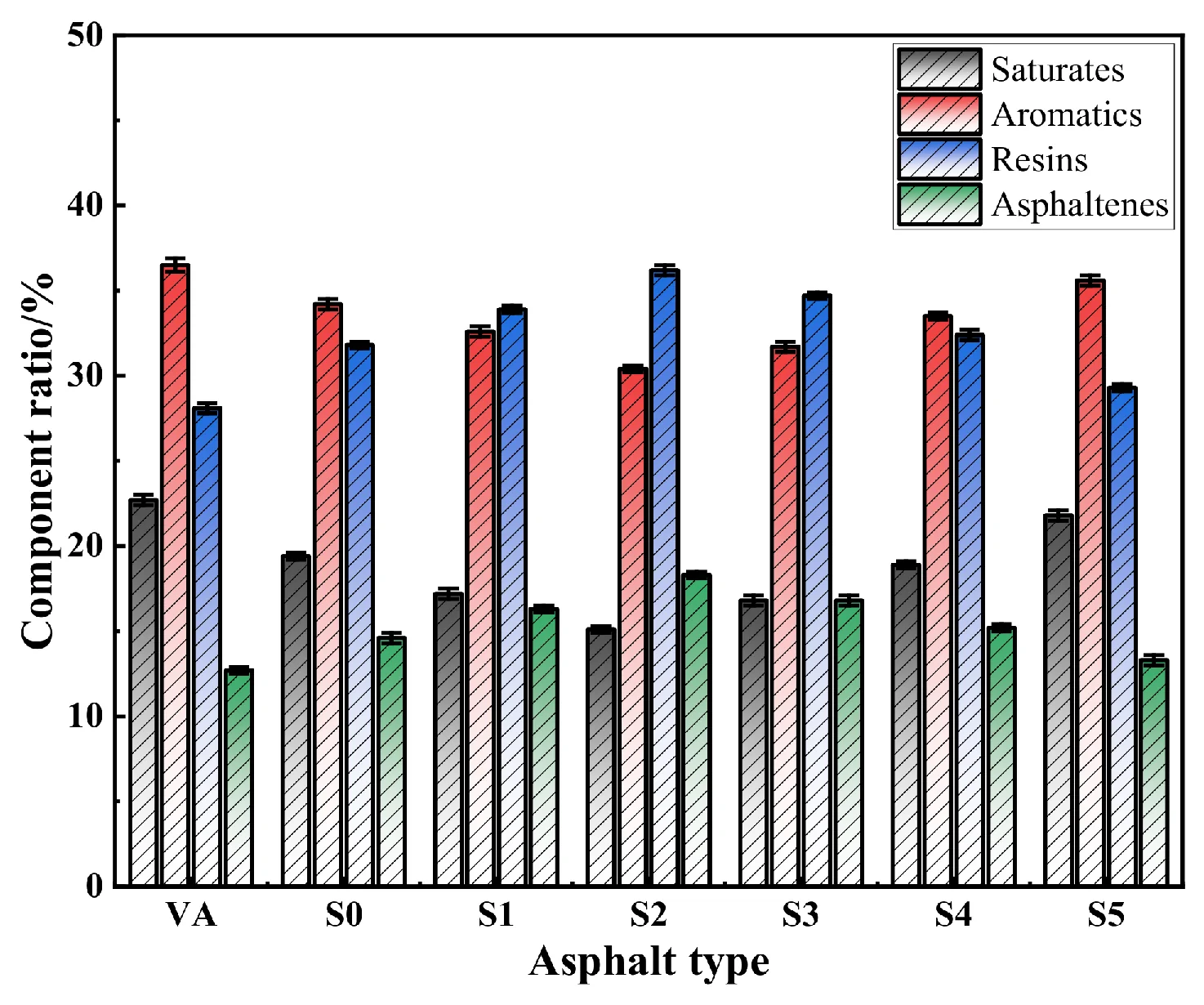
Variation patterns of components in several asphalts.

Combining thin-layer chromatography with flame ionization detection, the TLC-FID test separates asphalt fractions based on differences in component polarity. It quantitatively measures the mass proportion of four asphalt fractions: saturates, aromatics, resins and asphaltenes, and accurately characterizes the reconstruction effect of modification on asphalt colloidal composition. Therefore, TLC-FID tests were carried out on all asphalt specimens to analyze the variation in chemical fractions, and the test results are displayed in Figure 8.

It can be seen from Figure 8 that the mass fractions of saturates and aromatics generally decrease first and then increase, while the mass fractions of resins and asphaltenes rise first and then decline. The extreme values of all four fractions appear in specimen S2. Comparison of fraction data between VA and S0 reveals that after the addition of polymer modifier, the saturate and aromatic contents of S0 drop markedly relative to VA, accompanied by synchronous growth in resin and asphaltene proportions. This fraction variation indicates that long-chain modifier molecules carry polar groups, which can capture free light fractions in the system via intermolecular forces, reduce the content of freely migrating small molecules, indirectly raise the relative proportion of heavy cementitious fractions, and preliminarily enhance the stability of asphalt colloidal structure.

When fillers are continuously incorporated to prepare S1 and S2, the proportion of light fractions further declines while heavy fractions keep increasing, reaching the maximum variation amplitude at S2. Abundant silanol active sites distributed on filler surfaces possess strong adsorption capacity, which can further immobilize saturates and aromatics inside the system. A large amount of light fractions adhere to filler particle surfaces and lose the ability of free migration. The continuous colloidal core phase composed of resins and asphaltenes accounts for a remarkably higher proportion, forming a denser and more stable colloidal dispersion system. After normalization to pure organic asphalt matrix excluding nano-SiO_2_ solid filler, saturate and aromatic mass fractions gradually rebound for S3~S5, while resin and asphaltene proportions continuously decrease. The core cause of this phenomenon is severe particle agglomeration under excessive filler dosage. Agglomerated structures drastically reduce the overall specific surface area, which significantly weakens the filler’s capacity to adsorb light fractions. The previously restrained light fractions desorb and re-disperse throughout the system. Meanwhile, filler agglomerates split the continuous cementitious network formed by resins and destroy the intact structure of colloidal cores, leading to continuous deterioration of asphalt colloidal stability. At S5, the mass fractions of four fractions are highly close to those of VA.

Based on quantitative fraction results from TLC-FID, polymer modifiers and fillers at an appropriate dosage exert synergistic adsorption effects, which reduce free light fractions in the system, increase the proportion of heavy cementitious fractions and optimize the colloidal composition structure of asphalt. Excess filler dosage triggers particle agglomeration and drastically attenuates adsorption capacity, resulting in massive dissociation of light fractions and damage to colloidal integrity. This explains the existence of an optimal filler dosage range for the modified system from the perspective of microscopic fraction composition. The enrichment of resin and asphaltene heavy fractions and immobilization of light fractions at the optimal dosage construct a dense colloidal system with strong stress relaxation ability. Such component redistribution balances high-temperature permanent deformation resistance from MSCR tests and low-temperature crack resistance characterized by BBR creep stiffness and m-value.

At the nano-SiO_2_ dosage of 2%, uniform particle dispersion without agglomeration enables surface silanol groups to sufficiently adsorb saturates and aromatics and facilitate the association of small asphalt molecules into macromolecules, yielding the maximum proportion of resins and asphaltenes. The integrated system consisting of SBS elastic networks and inorganic rigid nano-silica skeletons effectively restricts molecular slippage and stress concentration, synchronously optimizing high-temperature rutting resistance, low-temperature crack resistance and fatigue performance. At a lower dosage of 1%, insufficient utilization of the filler’s specific surface area limits light fraction adsorption and macromolecular aggregation, forming an incomplete rigid skeleton that only slightly elevates rheological properties. When the nano-SiO_2_ dosage exceeds 2%, severe particle agglomeration drastically reduces available specific surface area and triggers light fraction desorption; agglomerates break the continuous colloidal phase and create abundant weak interfaces, causing aggravated stress concentration, retarded high-temperature performance improvement, increased low-temperature brittleness and shortened fatigue life.

## 4. Conclusions

In this study, VA was selected as the base material with a fixed SBS dosage of 4.5%. Six groups of asphalt specimens were prepared by adding nano-SiO_2_ at gradient dosages ranging from 0% to 5%. A series of physical property tests, macroscopic rheological tests including temperature sweep, MSCR, LAS and BBR, as well as microscopic characterization tests such as FTIR, GPC and TLC-FID were carried out. The rheological evolution law and microscopic interaction mechanism of asphalt synergistically modified by nano-SiO_2_ and SBS were clarified. The main conclusions are summarized as follows:

(1) The results of conventional physical performance tests reveal that with the increase in nano-SiO_2_ dosage, the penetration of asphalt decreases continuously while the softening point rises steadily, which continuously improves the high-temperature stiffness of asphalt. The low-temperature ductility shows a trend of increasing first and then decreasing, and reaches the maximum value when the nano-SiO_2_ dosage is 2%. At this dosage, the high-temperature stability and low-temperature plasticity of asphalt achieve balanced coordination.
(2) The organic elastic network formed by SBS and the inorganic rigid skeleton constructed by nano-SiO_2_ exert synergistic modification effects. Asphalt with 2% nano-SiO_2_ possesses the optimal G*, G*/sinδ and R, as well as the minimum Jnr, showing outstanding rutting resistance under high-temperature and heavy load. Meanwhile, asphalt at this mixing proportion presents the longest fatigue life under multiple strain levels, a slight increase in creep stiffness S and a relatively high creep rate m, which balances excellent fatigue durability and low-temperature stress relaxation capacity. Excessively high nano-SiO_2_ dosage leads to particle agglomeration and stress concentration, which simultaneously weakens the comprehensive performance of asphalt including high-temperature permanent deformation resistance, medium-temperature fatigue resistance and low-temperature crack resistance.
(3) Only physical interactions exist throughout the compound modification process of SBS and nano-SiO_2_, without chemical reactions or generation of new characteristic functional groups. Nano-SiO_2_ at a dosage of 2% can fully adsorb light fractions such as saturates and aromatics inside asphalt, promote the aggregation of small asphalt molecules and increase the proportion of macromolecular components, moderately increase the heavy-fraction content of asphalt, and form a stable and dense colloidal structure. Excessive filler dosage causes severe particle agglomeration, which greatly reduces the specific surface area of the material and significantly weakens its adsorption capacity for light fractions, thus destroying the continuous cementitious network. The molecular weight distribution and four-component composition tend to be consistent with base asphalt. This evolution law of microstructure is the fundamental internal mechanism accounting for the variation in macroscopic pavement performance of composite modified asphalt.

From an engineering perspective, this study provides a clear optimal nano-SiO_2_ dosage of 2% for 4.5% SBS-modified asphalt and establishes a multi-scale evaluation framework combining conventional binder tests, full-spectrum rheological characterization and quantitative microscopic analysis. The synergistic modification mechanism revealed in this work avoids the high construction viscosity and extra material cost caused by simply increasing SBS content, offering reliable theoretical guidance and formulation reference for heavy-load expressway and high-modulus pavement construction. Nevertheless, the present research has several limitations: only 90# neat asphalt is adopted as the base binder without verifying the adaptability of this composite modification system for asphalt with different penetration grades; all tests are conducted on asphalt binders at laboratory scale, while the corresponding mixture performance and long-term pavement service behavior remain unexamined; in addition, coupled aging effects including ultraviolet radiation, high-temperature and water erosion are not taken into consideration. For follow-up research, further investigations can be carried out in the following directions: expanding raw material systems with various base asphalt grades; conducting mixture performance tests such as wheel tracking and low-temperature bending beam tests; exploring the evolution of rheological and microstructural properties under multi-factor aging environments; modifying the surface of nano-SiO_2_ to mitigate particle agglomeration and broaden the effective dosage range; and implementing field paving trials to monitor the long-term service performance of composite modified asphalt pavements.

## Figures and Tables

**Table 1:** Basic Properties of Asphalt.

Test Index	Unit	Test Standard	Test Result	Technical Requirement
Penetration	0.1 mm	JTG E20-2011 T0604	86.6	60~80
Softening Point	°C	JTG E20-2011 T0606	45.6	≥44
Ductility	cm	JTG E20-2011 T0605	>100	≥100

**Table 2:** Basic Technical Parameters of SBS Modifier.

Test Index	Unit	Parameter Value
Block Ratio (Styrene/Butadiene)	-	30/70
Number Average Molecular Weight	g/mol	12 × 10^4^
Melt Flow Rate (190 °C, 5 kg)	g/10 min	0.7
Tensile Strength	MPa	≥18.0
Elongation at Break	%	≥700
Shore A Hardness	-	85~90

**Table 3:** Technical Indicators of Nano-SiO_2_.

Test Item	Unit	Parameter
Primary Average Particle Size	nm	12
BET Specific Surface Area	m^2^/g	200 ± 25
SiO_2_ Purity	%	≥99.8
pH Value (4% Aqueous Dispersion)	-	3.6~4.5
Bulk Density	g/L	50
Moisture Content	%	≤1.0
Surface Hydroxyl Content	mmol/g	2

**Table 4:** Sample Grouping and Working Condition Design of Asphalt.

Number	Sample Type	SBS Dosage/%	Nano-SiO_2_ Dosage/%
VA	Virgin Asphalt	0	0
S0	single-SBS-modified Asphalt	4.5	0
S1	SBS/Nano-SiO_2_ Composite Modified Asphalt	4.5	1
S2	SBS/Nano-SiO_2_ Composite Modified Asphalt	4.5	2
S3	SBS/Nano-SiO_2_ Composite Modified Asphalt	4.5	3
S4	SBS/Nano-SiO_2_ Composite Modified Asphalt	4.5	4
S5	SBS/Nano-SiO_2_ Composite Modified Asphalt	4.5	5

## Data Availability

The original contributions presented in this study are included in the article. Further inquiries can be directed to the corresponding authors.
